# Economic Burden of Chronic Obstructive Pulmonary Disease and Lung Cancer Between 2000 and 2015 in Saskatchewan: Study Protocol

**DOI:** 10.2196/31350

**Published:** 2022-03-04

**Authors:** Erika Dianne Penz, Benjamin John Fenton, Nianping Hu, Darcy Marciniuk

**Affiliations:** 1 Division of Respirology, Critical Care and Sleep Medicine University of Saskatchewan Saskatoon, SK Canada; 2 Respiratory Research Centre University of Saskatchewan Saskatoon, SK Canada; 3 Saskatchewan Health Quality Council Saskatoon, SK Canada

**Keywords:** lung cancer, COPD, chronic obstructive pulmonary disease, productivity loss, years of life lost, premature years of life lost, working years lost, economic burden of disease, lung disease, health economics, Stats Canada, epidemiology, pulmonary disease, pulmonary health, disease burden

## Abstract

**Background:**

Chronic obstructive pulmonary disease (COPD) and lung cancer are both detrimental diseases that present great burdens on society. Years of life lost (YLL), premature years of life lost (PYLL), working years lost (WYL), and productivity loss are all effective measures in identifying economic burden of disease.

**Objective:**

We propose a population-based study to analyze comprehensive provincial cohorts of Saskatchewan residents with COPD, lung cancer, and combined COPD and lung cancer in order to identify the burden these diseases present.

**Methods:**

Saskatchewan residents over the age of 35 years who had COPD, lung cancer, or both, between January 1, 2000, and December 31, 2015, will be identified and used in this study. Data for analysis including age, gender, and date of death, alongside Statistics Canada income estimates, will be used to estimate productivity loss and WYL. Statistics Canada life tables will be used to calculate YLL and PYLL by subtracting the patients’ ages at death by their life expectancies, adjusted using sex and age at death. We will link the Saskatchewan cancer registry with Saskatchewan health administrative databases to create three cohorts: (1) COPD; (2) lung cancer; and (3) COPD and lung cancer. Individuals with lung cancer will be identified using ICDO-T (International Classification of Diseases for Oncology-Topography) codes, and those with COPD will be defined and identified as individuals who had at least 1 visit to a physician with a diagnosis of COPD or 1 hospital separation with a diagnosis of COPD. Those without a valid health care coverage for a consecutive 12 months prior to the first diagnostic code will be excluded from the study. Those with a combined diagnosis of COPD and lung cancer will be identified as individuals who were diagnosed with COPD in the 12 months following their lung cancer diagnosis or anytime preceding their lung cancer diagnosis.

**Results:**

As of April 2021, we have had access to all relevant data for this study, have received funding (January 2020), and have begun the preliminary analysis of our data set.

**Conclusions:**

It is well documented that COPD and lung cancer are both destructive diseases in terms of YLL, PYLL, WYL, and productivity loss; however, no studies have been conducted to analyze a cohort with combined COPD and lung cancer. Understanding the economic burden associated with each of our 3 cohorts is necessary in understanding and thus reducing the societal impact of COPD and lung cancer.

**International Registered Report Identifier (IRRID):**

RR1-10.2196/31350

## Introduction

Chronic obstructive pulmonary disease (COPD) and lung cancer both pose great burdens on society in terms of years of life lost (YLL), premature years of life lost (PYLL), absenteeism, and productivity loss. Lung cancer is the leading cause of cancer mortality in Canada [1], and COPD was the third leading cause of death worldwide in 2010 [2]. Both diseases are predominant in older age groups, with the majority of patients receiving diagnosis in their sixties [3,4].

Due to the impact that COPD and lung cancer have on the respiratory system and symptoms, individuals are often greatly affected in their ability to work, and many withdraw from the workforce early due to disability or death [5]. It has been found that lung cancer is a significant contributor to YLL, accounting for nearly as many YLL as colon, breast, and prostate cancers combined [6]. COPD has also been shown to account for 1.8% of all deaths in Poland between 1999 and 2014. In addition, the typical patient with COPD lost roughly 14.5 years of life due to the disease [7]. The authors concluded that since smoking remains the leading cause of COPD, public education on the risks of smoking could have a considerable effect on reducing the burden of COPD in Poland [7]. A study examining mortality trends in Ontarians with COPD from 1996 to 2012 found that PYLL due to COPD decreased over the period, while COPD prevalence increased. The authors posited that the decrease in PYLL due to COPD is likely driven by improvements in both cardiovascular prognosis and COPD management. It also demonstrated that the age-sex standardized mortality rates for lung cancer in the COPD population decreased between 1996 and 2009, although the absolute number of people with COPD dying of lung cancer increased over the same period [8]. Despite the positive trend in YLL over this time period, the authors reported that PYLL for individuals with COPD were nearly 5 times higher than that of the non-COPD population.

Although YLL is a measure of the impact of a disease on death in a population, productivity loss is another important measure of the impact of a disease, reflected in measures such as absenteeism and WYL due to disability or premature death [9-11]. It has been found that, of the total direct and indirect costs associated with COPD, productivity loss accounts for nearly one-third of total associated costs [11]. A study conducted in Spain in 2019 found that lung cancer was responsible for 60,846 WYL and €13.1 billion (US $14.8 billion) in productivity loss between the years 2008 and 2017 [9]. However, the impact of a combined diagnosis of COPD and lung cancer, a common clinical occurrence, on YLL or productivity loss has not been well described. Given factors such as symptom burden and potentially increased comorbidities from shared risk factors, one would intuitively expect the true burden to be even greater. It has been established that COPD is a risk factor for lung cancer, even after controlling for smoking [12]. Additionally, a high proportion of patients with lung cancer have concomitant COPD [13].

Saskatchewan is a Canadian prairie province with a population of 1.098 million, covering 588,000 square kilometers with a population density of 1.9 people per square kilometer [14]. In 2017, smoking prevalence in Saskatchewan was 17.8% compared with 16.9% in 2015 and was above the national average of 15.1% [15]. Among youth, Saskatchewan has roughly double the national smoking rates [15]. Due to a growing and aging population, the number of cases of COPD and lung cancer continues to rise, and as a consequence, both diseases are significant public health concerns. We believe the results from this work would be both interesting and informative to many beyond this geographical area. Identifying the extent of COPD and lung cancer’s economic burden on Saskatchewan is crucial in understanding these diseases and what actions are appropriate in reducing their impact on society [16].

The objective of this study is to investigate the economic burden associated with COPD and lung cancer by estimating YLL, PYLL, WYL and productivity loss among a cohort of COPD and lung cancer patients in Saskatchewan diagnosed between the years 2000 and 2015.

## Methods

### Database and Data Linkage

We will link the Saskatchewan cancer registry with Saskatchewan health administrative databases to create three cohorts of patients: (1) COPD; (2) lung cancer; and (3) COPD and lung cancer between January 1, 2000, and December 31, 2015. The Saskatchewan cancer registry contains, among other variables, unique health services number, date of birth (year/month), date of diagnosis, date of death, primary cause of death, ICDO-T (International Classification of Diseases for Oncology-Topography) and ICDO-M (International Classification of Diseases for Oncology-Morphology) code of the primary tumor, and TNM overall stage. eHealth Saskatchewan is a government agency mandated to manage the information technology needs of the Saskatchewan Health Authority, which is responsible for and is the sole provider of government “single-payer” health care delivery in the province. It collects, combines, stores, and manages the electronic health records of Saskatchewan residents. Via eHealth Saskatchewan, the cancer registry data will be linked with the following Saskatchewan health administrative databases: Person Health Registry System, Discharge Abstracts Databases, and Physician Services Claims File. The Person Health Registry System contains unique health services number (encrypted), year of birth, sex, dates of health insurance coverage, and reason for termination. The Discharge Abstracts Databases contain health services number (encrypted), year and month of birth, date of admission, date of discharge, and discharge diagnosis (International Classification of Diseases 9th Revision [ICD-9] or 10th Revision [ICD-10], all fields). The Physician Services Claims file contains health services number (encrypted), diagnostic code (ICD) associated with service, service code, and date of service.

### Case Definitions

#### Lung Cancer

Using data from the Saskatchewan Cancer Agency registry, individuals 35 years of age or older diagnosed with small cell lung cancer (SCLC) or non–small cell lung cancer (NSCLC) between January 1, 2000, and December 31, 2015, will be included in the study. NSCLC and SCLC will be identified using the ICDO-T codes (Tables 1 and 2 in Multimedia Appendix 1). Individuals who were diagnosed at autopsy or death, who did not reside in Saskatchewan at the time of diagnosis, or who had a prior or subsequent cancer (except basal cell carcinoma or squamous cell carcinoma skin cancer) will be excluded.

#### Chronic Obstructive Pulmonary Disease

Individuals with COPD will be identified from Saskatchewan health administrative databases. COPD will be defined as an individual aged 35 years and older having at least 1 visit to a physician with a diagnosis of COPD in the first diagnostic field, or 1 hospital separation with a diagnosis of COPD in any diagnostic field, coded by the ICD-9-CM 491-492 (chronic bronchitis, emphysema), 496 (chronic airway obstruction, not otherwise specified), or ICD-10-CA J41-44 (chronic bronchitis) [17] until December 31, 2015. Individuals meeting these criteria prior to January 1, 2000, will be considered prevalent COPD cases while individuals meeting these criteria between January 1, 2000, and December 31, 2015, will be considered incident cases. Those without valid health care coverage for a consecutive 12 months prior to the first diagnostic code will be excluded from the study.

#### Chronic Obstructive Pulmonary Disease and Lung Cancer

All individuals identified with lung cancer between 2000 and 2015 who also meet the criteria for COPD in the 12 months following or anytime preceding their lung cancer diagnosis will be classified as a third cohort of individuals with both diagnoses.

### Population Cohorts and Time Frame

The COPD cohort will be as follows: adults aged 35 years or older diagnosed with COPD as of January 1, 2000, plus all new COPD cases identified until December 31, 2015.

The lung cancer cohort will be as follows: Adults aged 35 years or older diagnosed with NSCLC or SCLC between January 1, 2000, and Dec 31, 2015.

The COPD and lung cancer cohort is as follows: Adults aged 35 years or older diagnosed with NSCLC or SCLC between January 1, 2000, and Dec 31, 2015, who also meet the criteria for COPD in the 12 months following or anytime preceding a lung cancer diagnosis.

All individuals will be followed to the end of study (December 31, 2018), end of coverage, or death, whichever occurs first.

### Outcomes

#### Death

Date of death will be obtained from 2 sources, the cancer registry for the lung cancer and combined lung cancer and COPD cohorts, and the Saskatchewan personal health registry database for the COPD cohort. These data are updated quarterly from eHealth.

#### Years of Life Lost

YLL will be calculated for each cohort by subtracting the subject’s age at death from their Saskatchewan age-specific and sex-specific life expectancy in their year of death. Life expectancy will be obtained from published life tables through Statistics Canada [18]. Average YLL will be calculated for all 3 cohorts by dividing YLL by the number of individuals who died in each cohort. YLL and average YLL will be stratified by gender, as well as type and stage of lung cancer.

#### Premature Life Years Lost

Premature life years lost (PYLL) will be reported for each cohort including only those individuals who died before their expected age-adjusted and sex-adjusted life expectancy at the time of death.

Average PYLL will be calculated for each cohort by dividing total PYLL by the number of individuals in each cohort who died prematurely. These results will be stratified by gender as well as type and stage of lung cancer.

#### Working Years Lost and Productivity Loss

Working years lost (WYL) will be calculated by subtracting the date of death from the birth date in the expected retirement year. If the value is positive, this is recorded as the WYL. If the value is negative, this is recorded as zero.

Productivity loss will be calculated by multiplying an individual’s WYL, if positive, by their age-adjusted and sex-adjusted income using Saskatchewan-specific income statistics from Statistics Canada [19].

### Base Case Model and Scenario Analysis

The base case model will use an expected retirement age of 65 years for both men and women for calculation of WYL. Median income will be used to calculate productivity loss. Scenario analyses will be conducted to explore difference in WYL and productivity loss according to different age at retirement as well as the use of mean income statistics from Statistics Canada. The scenarios to be analyzed are as follows: scenario 1—WYL based on retirement at age 63 years for men and 61 years for women [20]; scenario 2—WYL based on retirement at 55 years of age; and scenario 3—WYL based on retirement at 70 years of age.

### Labor Force Participation

To adjust the estimated productivity loss for each cohort because not all individuals are employed in the labor force, we will incorporate age-specific and sex-specific labor force participation rates from Statistics Canada into our outcomes for each cohort (ie, expected WYL and expected productivity loss).

### Inflation

We will report all productivity loss estimates in 2018 Canadian dollars. In the case of WYL prior to 2018, age-specific and sex-specific incomes will be obtained directly from Statistics Canada, which are provided in inflation-adjusted 2018 Canadian dollars. In the case of WYL after 2018, income will be inflated based on average age-specific and sex-specific growth rates calculated over the time period of 2000-2018.

### Statistical Methods

#### Productivity Loss Model Structure

In order to identify lost productivity due to premature death, we must assess the affected individuals’ lost incomes. Lost productivity will be measured through 2 specific metrics, which are productivity loss and WYL. We will report averages and totals, as well as the expected values for both metrics. The results will be stratified by gender and stage of lung cancer (for lung cancer and combined groups). In the first and last years of lost income, partial WYL will be calculated.

#### Formulas

We will calculate working years lost, year at expected retirement, total productivity loss, and expected total productivity loss. The total productivity loss formula will sum income adjusted using sex (s) and age (a) for years between year at death and year at expected retirement, while the expected total productivity loss formula will adjust for sex-specific and age-specific labor force participation rates as well. The formulas we will use are as follows:

Working years lost = expected retirement age - age at death;

YAR = working years lost + calendar year at death;

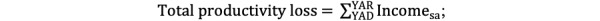





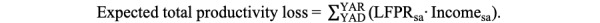



Where YAD is years between calendar year at death, YAR is calendar year at expected retirement, and LFPR is labor force participation rates.

#### Partial Years Correction

The time between date of death and December 31 in the year of death, and the time between January 1 and the individual’s birthday in the expected year of retirement will be calculated as partial years in order to estimate productivity loss more precisely. For example, if someone died on December 1, 2015, the WYL and income attributed to that year would be equal to 30/365 for WYL and (30/365) multiplied by age-adjusted and sex-adjusted 2015 median income for productivity loss in 2015. All years between year at death and year at retirement will be accounted as full WYL.

### Ethics Approval

Ethics exemption by the Research Ethics Board was received on May 31, 2017 (Bio 17-153).

## Results

As of April 2021, we have gained access to all relevant data for this study, have received funding from the Lung Association of Saskatchewan (January 2020), and have begun preliminary analysis of our data set.

## Discussion

### Comparison With Prior Work

This study’s aim is to estimate the economic burden, by way of YLL, PYLL, and productivity loss among COPD and lung cancer patients in Saskatchewan using comprehensive government health administrative and cancer registry data. Although prior work estimating indirect costs has been reported for COPD and lung cancer populations [2,9,11], there has not been a comparison between the 2 diseases over the same time period, from the identical general sample population for the extended time period we studied or describing indirect costs in a population of individuals with both diagnoses.

By understanding indirect costs associated with COPD and lung cancer through measures of PYLL, WYL, and productivity loss, these estimates can help inform policy makers, public health professionals, and the public to better understand the burden associated with lung disease experienced outside the health system. These costs are frequently missed or minimized even though they may be substantial to both the individual and the population. Moreover, from a governmental point of view, quantifying the magnitude of the economic impact of these diseases can assist policy makers when deciding on priorities related to health service delivery (eg, expanding access to diagnostic services to enable early detection) and efforts to improve prevention strategies (eg, expanding smoking cessation programs).

According to previous literature, lung cancer and COPD are comparable in terms of average YLL and average PYLL, so we expect to find similar results in those measures [6,7]. Due to an expected larger number of patients diagnosed with COPD compared with lung cancer, the total YLL, total PYLL, and total productivity loss will be highest in the COPD group. The benefit of reporting the aggregate productivity loss as well as YLL for each disease will be to estimate the overall magnitude of economic costs over the study period. If the studies performed by Kim et al [11] and Darba and Marsa [9] are indicators of what to expect from our data, productivity loss due to these diseases could exceed CAD $1 billion (US $788 million). It is unclear what the economic costs associated with the combined COPD and lung cancer group will be relative to either diagnosis alone; however, we hypothesize that mean YLL (as well as PYLL), WYL, and productivity loss may be greater than either diagnosis alone.

### Limitations

Despite using sex-adjusted and age-adjusted average and median incomes for Saskatchewan residents from Statistics Canada for the productivity loss estimates in our study, total and average productivity loss will not be based on the individuals’ actual income levels; this is a limitation with our analysis. Another limitation is that productivity loss in our study does not take into account the time lost due to illness (as opposed to death), nor does it account for time away from work and the associated productivity loss attributed to caregivers. Lastly, life years and premature life years lost will be calculated using age-adjusted and sex-adjusted life tables from Statistics Canada in relation to death from any cause in each of the cohorts; therefore, YLL, and PYLL may not completely be attributed to COPD, lung cancer, or both diagnoses entirely.

## Appendix

Multimedia Appendix 1 International Classification of Diseases for Oncology Topography and Morphology definitions.

## Abbreviations

COPD: chronic obstructive pulmonary disease
ICD: International Classification of Diseases
ICDO-M: International Classification of Diseases for Oncology-Morphology
ICDO-T: International Classification of Diseases for Oncology-Topography
NSCLC: non–small cell lung cancer
PYLL: premature years of life lost
SCLC: small cell lung cancer
WYL: working years lost
YLL: years of life lost

## Availability of Data and Materials

The data sets analyzed during this study are not publicly available due to the terms and requirements of the data sharing agreements governing health administrative data access in Saskatchewan.
