# Supplementary material for: Economic Burden of Chronic Obstructive Pulmonary Disease and Lung Cancer Between 2000 and 2015 in Saskatchewan: Study Protocol
Source: JMIR Res Protoc. 2022 Mar 4;11(3):e31350. doi: 10.2196/31350 (PMC8933801; doi:10.2196/31350)
Supplement: Multimedia Appendix 1 [file resprot_v11i3e31350_app1.docx]

# Multimedia Appendix 1. International Classification of Diseases for Oncology Topography and Morphology definitions.

| Code | Diagnosis | Small cell (X) | Non-small cell (X) |
| --- | --- | --- | --- |
| C33.9 | Lung |  |  |
| C34.0 | Bronchus, main |  |  |
| C34.0 | Carina |  |  |
| C34.0 | Hilus |  |  |
| C34.0 | Main bronchus |  |  |
| C34.1 | Bronchus, upper lobe |  |  |
| C34.1 | Lingula |  |  |
| C34.1 | Upper lobe |  |  |
| C34.1 | Upper lobe, bronchus |  |  |
| 34.2 | Bronchus, middle lobe |  |  |
| C34.2 | Middle lobe |  |  |
| C34.2 | Middle lobe, bronchus |  |  |
| C34.3 | Bronchus, lower lobe |  |  |
| C34.3 | Lower lobe |  |  |
| C34.3 | Lower lobe, bronchus |  |  |
| C34.9 | Bronchiole |  |  |
| C34.9 | Bronchogenic |  |  |
| C34.9 | Bronchus, NOS |  |  |
| C34.9 | Pulmonary, NOS |  |  |
| C34.9 | NOS |  |  |

**Table 1: Applicable ICD-O T-codes**

**Table 2: Applicable ICD-O M-codes**

| Code | Diagnosis | Small cell (X) | Non-small cell (X) |
| --- | --- | --- | --- |
| 8070/3 | Squamous cell carcinoma |  | S |
| 8052/3 | Papillary |  | A |
| 8084/3 | Clear cell |  | L |
| 8073/3 | Small cell | SC |  |
| 8083/3 | Basaloid |  | S |
|  |  |  |  |
| 8041/3 | Small cell carcinoma | SC |  |
| 8045/3 | Combined small cell carcinoma | SC |  |
|  |  |  |  |
| 8140/3 | Adenocarcinoma |  | A |
| 8255/3 | Adenocarcinoma, mixed subtype |  | A |
| 8550/3 | Acinar adenocarcinoma |  | A |
| 8550/3 | Papillary adenocarcinoma |  | A |
| 8250/3 | Bronchioloalveolar carcinoma |  | A |
| 8252/3 | Nonmucinous |  | A |
| 8253/3 | Mucinous |  | A |
| 8254/3 | Mixed nonmucinous and mucinous indeterminate |  | A |
| 8230/3 | Solid adenocarcinoma with mucin production |  | A |
| 8333/3 | Fetal adenocarcinoma |  | A |
| 8480/3 | Mucinous (“colloid”) carcinoma |  | A |
| 8470/3 | Mucinous cystadenocarcinoma |  | A |
| 8490/3 | Signet ring adenocarcinoma |  | A |
| 8310/3 | Clear cell adenocarcinoma |  | A |
|  |  |  |  |
| 8012/3 | Large cell carcinoma |  | L |
| 8013/3 | Large cell neuroendocrine carcinoma |  | L |
| 8013/3 | Combined large cell neuroendocrine carcinoma |  | L |
| 8012/3 | Basaloid carcinoma |  | L |
| 8082/3 | Lymphoepithelimoa-like carcinoma |  | L |
| 8014/3 | Clear cell carcinoma |  | L |
|  |  |  |  |
| 8560/3 | Adenosquamous carcinoma |  | AS |
|  |  |  |  |
| 8033/3 | Sarcomatoid carcinoma |  | R |
| 8022/3 | Pleomorphic carcinoma |  | R |
| 8032/3 | Spindle cell carcinoma |  | R |
| 8031/3 | Giant cell carcinoma |  | R |
| 8980/3 | Carcinosarcoma |  | R |
| 8972/3 | Pulmonary blastoma |  | R |
|  |  |  |  |
| 8000 | Neoplasm/tumor NOS |  | NOS |
| 8010 | Carcinoma NOS |  | NOS |
|  |  |  |  |
| 8550/3 | Acinar cell carcinoma https://codes.iarc.fr/code/3064 |  | A |
| 8240/3 | Carcinoid tumor |  | C |
| 8240/3 | Typical carcinoid |  | C |
| 8249/3 | Atypical carcinoid |  | C |
|  |  |  |  |
| 8430/3 | Salivary gland tumors |  | R |
| 8430/3 | Mucoepidermoid carcinoma |  | R |
| 8200/2 | Adenoid cystic carcinoma |  | R |
| 8562/3 | Epithelial-myoepithelial carcinoma |  | R |
|  |  |  |  |
| 8246/3 | Neuroendocrine carcinoma | SC |  |
| 8043/3 | Fusiform cell carcinoma | SC |  |
|  |  |  |  |
| 8251/3 | Alveolar adenocarcinoma |  | A |
| 8481/3 | Mucin-Producing adenocarcinoma |  | A |
| 8260/3 | Papillary adenocarcinoma |  | A |
| 8123/3 | Basaloid carcinoma |  | S |
| 8071/3 | Squamous Cell CA, keratinizing, NOS |  | S |
| 8072/3 | Squamous Cell CA, large cell, non-keritinizing, NOS |  | S |

## Histology Groupings

Non-small cell lung carcinoma (NSCLC)

Adenocarcinoma (A)

Squamous cell carcinoma (S)

Large cell carcinoma (L)

Adeno-squamous (AS)

Rare subtypes including sarcomatoid, spindle, blastoma, etc..(R)

Carcinoid (C)

Not otherwise specified (NOS)

Small cell lung carcinoma (SC)
